# Age-Stratified Heterogeneity of Brucellosis Awareness and Knowledge Among Hospital-Attending Adults in an Endemic Turkish Province: A Single-Center Cross-Sectional KAP Study

**DOI:** 10.3390/tropicalmed11070185

**Published:** 2026-07-06

**Authors:** Enes Dalmanoğlu, İrem Sakarya, Ali Osman Yıldız, Muhammed Taha Özügüzel, Çiğdem Işık, Ahmet Enes Kaya, Yasin Uslu, Vedat Elgün, Muhammed Enes Geylani

**Affiliations:** 1 Department of Infectious Diseases and Clinical Microbiology, Faculty of Medicine, Balıkesir University, 10145 Balıkesir, Türkiye; 2 Faculty of Medicine, Balıkesir University, 10145 Balıkesir, Türkiye

**Keywords:** brucellosis, health knowledge, attitudes, practice, health literacy, cross-sectional studies, age factors, zoonoses, one health

## Abstract

Background: Human brucellosis is a widespread zoonotic febrile illness in livestock-rearing endemic regions, including Türkiye (pooled human seroprevalence approximately 4.5%). Age-stratified item-level knowledge profiles paired with information-source patterns are rarely reported in adult populations. We aimed to describe age-stratified brucellosis knowledge and information-source ecosystems in a hospital-attending adult sample from an endemic Turkish province. Methods: A hospital-based cross-sectional survey enrolled 397 adults in Balıkesir, Türkiye. A 17-question instrument assessed demographics, prior awareness, a 26-item composite knowledge score among aware respondents, and information sources. Item-level recognition was compared across four age strata, with multivariable logistic regression identifying independent predictors. Results: Of 397 adults (mean age 36.1 years; 62.7% male), 233 (58.7%) reported prior awareness, increasing with age (51.8% in 18–29 vs. 74.5% in ≥60 years; *p* < 0.001). Among aware respondents, composite knowledge declined with age (mean 8.52 vs. 5.94; *p* < 0.001). Raw dairy transmission and treatment availability were uniformly recognized (>80% across all strata). Recognition of clinical symptoms (fever 68.9% vs. 34.3%; *p* = 0.005) and veterinary signs (decreased milk yield 36.9% vs. 8.6%; *p* = 0.001) was substantially lower in older respondents. Internet citation declined with age (41.7% to 17.1%), while older respondents relied more on interpersonal networks. Conclusions: Brucellosis knowledge was not uniformly distributed across the adult age spectrum. Dominant transmission and treatment messages were near-universally recognized, while clinical-symptom and veterinary-sign recognition showed substantial age-related deficits, accompanied by generational divergence in information-source ecosystems. These findings suggest that age-tailored, channel-specific reinforcement of less-recognized clinical and veterinary knowledge, delivered through trusted healthcare-worker channels, may strengthen brucellosis education in endemic Turkish settings. Community-based replication is required before broader policy translation.

## 1. Introduction

Recognized by the World Health Organization as a priority neglected zoonotic disease and one of the world’s most widespread bacterial zoonoses, human brucellosis exemplifies the One Health interface between human, livestock, and environmental health, with sustained transmission concentrated in livestock-rearing endemic regions of low- and middle-income countries [1]. Brucellosis is the most prevalent bacterial zoonosis globally, with a recently revised conservative estimate of approximately 2.1 million annual human cases worldwide [2], and remains endemic across much of the Mediterranean basin, the Middle East, Central Asia, sub-Saharan Africa, and parts of Latin America [3]. In Türkiye, the disease has been endemic for decades; a 2024 nationwide systematic review and meta-analysis estimated a pooled human seroprevalence of approximately 4.5% in the general population, rising to 8.0% in rural areas and 9.9% in high-risk occupational groups, with the highest regional rates observed in Central East Anatolia [4]. Community-based seroprevalence studies in Western Anatolian provinces have documented rates ranging from 2.9% to 8.5% [5], confirming the ongoing endemicity in the Marmara region, in which Balıkesir is situated. Recent provincial-level investigations continue to document elevated seropositivity in eastern Anatolian provinces, with a 2023 multicenter study in Hakkari reporting a *Brucella*-Coombs-confirmed seroprevalence of 3.2% among 12,742 screened patients [6]. A 2024 multicenter clinical case series of 297 confirmed brucellosis patients in Türkiye documented that the majority of rural cases were farmers and that raw dairy consumption was the principal exposure route [7]. Despite three decades of focused vertical animal vaccination programs and public-health communication directed at the unpasteurized dairy transmission route, human incidence has remained essentially stable, suggesting that further control gains will require strategies more sophisticated than simple message repetition [8,9]. Effective brucellosis control ultimately depends on a One Health approach integrating human, animal, and environmental sectors, given that the disease reservoir lies primarily in livestock populations [10].

Knowledge-attitudes-practices (KAPs) surveys have served as the principal instrument for evaluating community-level brucellosis literacy for several decades [11,12]. These surveys conventionally aggregate multiple knowledge items into a composite score and report mean scores across demographic strata. While useful for population-level surveillance, this aggregation conceals item-level heterogeneity: it cannot distinguish between scenarios in which all knowledge elements decline uniformly across an age gradient versus scenarios in which a subset of dominant messages has reached high recognition, while ancillary domains remain less developed. The distinction matters for programmatic considerations because a uniform deficit might call for broader exposure to the same messages, whereas a non-uniform pattern might call for a reallocation of communication effort toward less-recognized domains. Recent KAP studies of brucellosis-confirmed patients in Xinjiang, China [13] and livestock farmers and meat handlers in Saudi Arabia [14] have begun to disaggregate knowledge by occupational and behavioral subgroups. To our knowledge, however, item-level age-stratified analysis of brucellosis knowledge paired with information-source mapping has not been previously reported in an adult Turkish hospital-attending population using a single descriptive cross-sectional framework with FDR-corrected inference, which is the specific descriptive niche of the present study.

An age-stratified heterogeneity pattern, if present, has direct implications for intervention design. If a dominant message has reached high recognition across all demographic groups, further intensification of that same message may yield diminishing incremental returns; resources may instead be redirected toward less-recognized knowledge domains where measurable gains are still achievable. Conversely, if generational differences in information-source ecosystems coexist with item-level differences, then channel-specific delivery strategies become a complementary lever [15,16]. Pairing item-level knowledge analysis with information-source mapping has not been systematically performed in the Turkish brucellosis literature, despite the policy salience of the question.

We conducted a hospital-based cross-sectional survey of brucellosis knowledge and information-source use in Balıkesir, an endemic Turkish province, with three pre-specified objectives: first, to characterize age-stratified brucellosis awareness and item-level knowledge with FDR-corrected inference distinguishing stable from differentially distributed items; second, to examine information-source use across age strata, testing whether generational divergence in channel ecosystems coexists with item-level knowledge differences; and third, to quantify the contribution of differential information-source use to the age-related composite-knowledge gradient using a covariate-adjustment framework.

To complement the principal item-level analysis, two exploratory summary tools were applied: a Communication Efficiency Index (CEI) integrating awareness breadth and knowledge depth, and a Three-Tier Knowledge Classification organizing item-level findings into descriptive categories.

## 2. Materials and Methods

### 2.1. Study Design, Setting, and Participants

**Figure 1:**
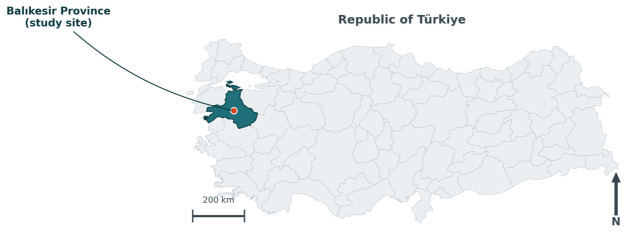
Geographical location of the study area. Balıkesir Province (highlighted), in the Southern Marmara region of northwestern Türkiye, is endemic to human brucellosis. The marker indicates Balıkesir city, the location of the tertiary referral hospital at which the cross-sectional survey was conducted. Provincial boundaries are shown for all 81 provinces of Türkiye.

We conducted a single-center, hospital-based, descriptive cross-sectional study at Balıkesir University Health Application and Research Hospital, a tertiary referral center serving Balıkesir Province (population approximately 1.25 million) in the Southern Marmara region of northwestern Türkiye (Figure 1). The study was designed and reported as a single-center hospital-based survey and is not intended to be representative of the general adult population of the province. The province combines urbanized coastal districts with livestock-dense interior districts supporting traditional small-ruminant husbandry and dairy production. Brucellosis is endemic throughout the province. The hospital receives outpatient referrals from the urban centers of the province and from the surrounding livestock-dense rural districts. Reporting followed the Strengthening the Reporting of Observational Studies in Epidemiology (STROBE) recommendations for cross-sectional observational studies [17].

Consecutive adults aged 18 years or older attending outpatient clinics between 15 January 2026 and 15 March 2026 were invited to participate by convenience sampling. We acknowledge that convenience sampling at a tertiary teaching hospital introduces selection bias toward urban, university-educated, and student populations; this important limitation is addressed in detail in the Section 4.3 and in our pre-specified sensitivity analysis. Inclusion criteria were age ≥18 years, current residence within Balıkesir Province, Turkish literacy sufficient to comprehend survey items, and written informed consent. Exclusion criteria were cognitive impairment, sensory or language barriers not accommodated by the interviewer reading, and refusal or incomplete consent. Of the 400 adults enrolled, 3 records lacked any age response and were excluded. A further 7 records contained out-of-range or implausible age entries in the data-entry sheet, arising from data-entry errors (including birth years inadvertently recorded in place of age); these were cross-checked against the original source survey documents and corrected to their true recorded ages, each of which fell within the valid adult range (≥18 years; corrected real values, not imputations). In the same validation step, a small number of records with a blank residence field (*n* = 5) or livestock-involvement field (*n* = 2) were similarly completed from the original source survey documents (verified values, not imputations). The final analytic sample comprised 397 participants with complete real age data. All participants were residents of one of the eight districts of Balıkesir Province, ensuring that the study reflects a single endemic province rather than a mixed geographic population.

Sample size (*n* = 384, inflated to 400 for incomplete responses) was calculated for prevalence estimation (95% confidence, ±5% precision, *p* = 0.50); a power calculation tailored to the primary item-level comparison was not pre-specified, which we acknowledge as a limitation. A post hoc analysis (G*Power 3.1 [18]) indicated that *n* = 397 provided at least 80% power to detect an 18-percentage-point between-stratum difference (Cohen h ≥ 0.37 [19]) by the Pearson χ^2^ test at α = 0.05, below the magnitude of the principal Tier 2 findings. The small ≥60-year stratum (*n* = 47) limits the precision of stratum-specific estimates for low-prevalence items.

### 2.2. Instrument and Knowledge Scoring

A structured 17-question instrument was adapted from prior Turkish and regional brucellosis surveys, including community-based seroprevalence studies that incorporated knowledge and exposure questionnaires across mid-Anatolia, central Anatolia, and Western Anatolia [20,21,22]. Section 1 (6 items) captured sociodemographic characteristics (age, sex, education, occupation, residence, livestock involvement). Section 2 (11 items) assessed prior awareness of brucellosis, operationalized as a single dichotomous (yes/no) response to a direct interviewer-administered question asking whether the respondent had ever heard of brucellosis (the question wording included the most common Turkish lay terms for brucellosis, such as Malta humması and Akdeniz humması, to maximize recognition); asking a pre-specified seven-domain knowledge battery, administered only to aware respondents (alternative names, transmission routes, human symptoms, animal signs, human vaccine availability, animal vaccine availability, and treatment availability); asking for personal or family history of disease; and asking for multi-select information sources (healthcare workers, veterinarians, family or neighbors, internet or social media, television or radio, newspaper or magazine, agricultural directorate, brochures or posters, personal experience). The completed STROBE checklist for cross-sectional studies is provided as a separate supplementary file.

The questionnaire was developed by adapting items from previously published brucellosis KAP studies in Türkiye and other endemic regional settings [20,21,22]. Item content covered disease synonyms, transmission routes, human and animal clinical signs, vaccination, and treatment, supplemented with an information-source item used in the covariate-adjustment analysis. The draft instrument was reviewed by infectious disease faculty and the broader research team for content relevance, clarity of Turkish phrasing, and cultural appropriateness; items were refined through iterative discussion until a consensus was reached. A small pre-deployment pilot conducted with outpatients (not included in the analytic sample) confirmed comprehensibility and acceptable administration time. We did not perform a formal psychometric validation study of the instrument prior to deployment; specifically, structured assessments of content, construct, and criterion validity, as well as test-retest reliability, were not conducted, and the questionnaire has not been validated against an external gold-standard measure of brucellosis knowledge. We acknowledge this as a methodological limitation. Within the main study sample, internal consistency of the 26-binary-item composite knowledge score was Cronbach α = 0.822 (Supplementary Table S1), supporting acceptable internal reliability of the composite measure; this post hoc estimate, however, does not substitute for prospective psychometric validation. Findings derived from the instrument should therefore be interpreted as descriptive of responses to this specific item battery in the present hospital-based sample, with appropriate caution regarding generalization to other populations or settings.

A 26-binary-item composite knowledge score (range 0–26) was constructed a priori by summing correct responses across seven content domains: alternative names of brucellosis (0–6 points across six commonly recognized lay terms, including Malta humması, Akdeniz humması, peynir hastalığı, mal hastalığı, koyun hastalığı, and yavru atma hastalığı), transmission routes (0–6 points across six routes: raw dairy, contaminated meat, cutaneous contact through cuts and wounds, inhalation/aerosol, breast milk transmission, and sexual contact), human symptoms (0–6 points across six symptoms: malaise, fever, night sweats, appetite loss, joint or muscle pain, and weight loss), animal signs (0–5 points across five signs: abortion, decreased milk yield, infertility, loss of breeding value, and testis inflammation), human vaccine availability (1 point if correctly identified as not available), animal vaccine availability (1 point if correctly identified as available), and treatment availability (1 point if correctly identified as available). To enable interpretable item-level inference and avoid the inflated multiple-comparison burden of testing all 26 individual binary items, a pre-specified subset of 16 substantive knowledge items considered most clinically and programmatically meaningful (the four most commonly cited transmission routes, all six human-symptom items, three principal animal signs, and the three vaccine/treatment items) constituted the FDR-corrected family for between-stratum item-level comparisons; the six alternative-name items were retained in the composite score for Cronbach reliability and CEI computation but were not entered into the FDR-corrected primary item-level analysis given their nomenclatural rather than substantive nature.

### 2.3. Data Collection

Eight trained investigators (authors İS, AOY, MTÖ, Çİ, AEK, YU, VE, and MEG) administered the survey face-to-face after a two-hour structured training in study protocol, interview technique, informed consent, and data privacy. Interviews were conducted in private hospital consultation areas. Each questionnaire received a sequential anonymous identifier; no personal identifiers were linked to responses. Completed paper forms were transcribed into a password-protected electronic database within 24 h and subjected to weekly inter-form consistency checks. Inter-interviewer consistency was monitored throughout data collection through weekly review meetings in which sample completed questionnaires were jointly reviewed and ambiguous response patterns were discussed.

### 2.4. Statistical Analysis

Analyses were performed in Python 3.11 (statsmodels 0.14, scipy 1.11, scikit-learn 1.3). Continuous variables are presented as mean ± standard deviation (SD) and median (interquartile range, IQR); categorical variables as *n* (%). Non-normality of the composite knowledge score was confirmed by the Shapiro–Wilk test (*p* < 0.001). Age was grouped into four strata (18–29, 30–44, 45–59, ≥60 years) following conventional epidemiological cut-points reflecting young-adult, middle-age, late-middle-age, and older-adult life stages used in prior Turkish public-health surveillance, specified before analysis.

The primary analysis compared item-level correct-recognition rates across the four age strata using the Pearson χ^2^ test. Items were classified a priori as stable (no significant difference across strata) or differentially distributed (significant difference) based on FDR-corrected α = 0.05. Secondary analyses included: (i) awareness of brucellosis by age using the Cochran–Armitage trend test; (ii) composite knowledge score by age using the Kruskal–Wallis test; (iii) Spearman rank correlation between continuous age and continuous knowledge score; and (iv) information-source citation rates by age stratum using χ^2^ tests with FDR correction.

Two pre-specified multivariable logistic regression models identified independent adjusted predictors of (a) awareness and (b) adequate knowledge among aware respondents. Adequate knowledge was defined a priori as the composite score ≥ the sample median, a threshold chosen before primary analysis given the absence of an externally validated cut-point in the brucellosis KAP literature; the resulting threshold was 7 of 26. We acknowledge that defining adequate knowledge using a sample-derived median threshold limits direct comparability with other studies and is acknowledged as a methodological choice; we therefore additionally report continuous composite scores (Section 3.2) and present the adequate-knowledge logistic regression primarily for consistency with prior KAP literature using similar dichotomization approaches. Candidate predictors entered simultaneously (rather than by automated selection) included age category, sex, education, residence, and livestock involvement in both models; family history of brucellosis was added only to the adequate-knowledge model because the family-history question was nested within the aware-respondent section of the questionnaire and is therefore not applicable as a predictor in the full-sample awareness model. Collinearity (variance inflation factor, VIF), discrimination (area under the receiver-operating-characteristic curve, AUC), and calibration (Hosmer–Lemeshow test) were assessed for both models. Adjusted odds ratios (aORs) are presented with Wald 95% confidence intervals (CIs) and 1000-iteration bootstrap percentile confidence intervals. A pre-specified sensitivity analysis re-fitted both models after excluding the 142 student participants. Diagnostic thresholds, the bootstrap random-seed specification, and events-per-variable ratios are detailed in Supplementary Note S1.

To explore information-source use as a possible explanatory channel, a secondary linear regression among aware respondents regressed composite score on continuous age alone and then jointly on age plus four information-source indicators (internet, family or neighbors, healthcare workers, veterinarian); the relative reduction in the age coefficient was calculated as [1 − (β_age | sources/β_age | crude)] × 100%. This is reported as a covariate-adjustment description rather than a formal mediation analysis, because cross-sectional data do not satisfy the temporal-ordering assumptions for causal mediation inference [23].

**Figure 2:**
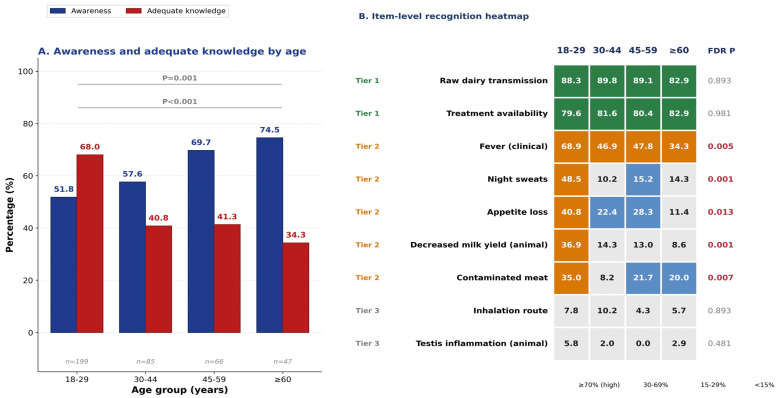
Age-stratified brucellosis awareness and item-level knowledge profile in 397 adults attending a tertiary teaching hospital in Balıkesir, Türkiye. (**A**) Awareness and adequate-knowledge percentages by age stratum, with within-bar value labels and cross-stratum significance brackets (Cochran–Armitage Z = 3.44 for awareness, *p* = 0.001; Z = −3.98 for adequate knowledge, *p* < 0.001). (**B**) Item-level recognition heatmap of nine representative knowledge items across four age strata among aware respondents (*n* = 233), color-coded by recognition rate (≥70% green, 30–69% orange, 15–29% blue, <15% gray). Tier classification (left margin) maps each item to the pre-specified Three-Tier Knowledge Classification. Right column shows Benjamini–Hochberg FDR-corrected χ^2^ *p* values; bold red indicates statistical significance (FDR *p* < 0.05). Tier 1 items (raw dairy transmission, treatment availability) show uniformly high and stable recognition across all strata, while Tier 2 items (fever, night sweats, appetite loss, decreased milk yield, contaminated meat) show substantial young-old recognition gradients. Tier 3 items (inhalation route, testis inflammation) show uniformly low and stable recognition.

Two pre-specified exploratory constructs summarized the joint breadth-depth pattern (Figure 2); neither has been externally validated. The Communication Efficiency Index (CEI) was defined as CEI(stratum) = P(aware | stratum) × E[composite score | aware, stratum]/26, with bootstrap 95% confidence intervals from 1000 resampling iterations. The Three-Tier Knowledge Classification was defined a priori from the FDR-corrected χ^2^ analysis of the 16 substantive items: Tier 1 (saturated: FDR *p* > 0.05 and recognition >70% in all strata), Tier 2 (differentially distributed: FDR *p* ≤ 0.05), and Tier 3 (stable-low: FDR *p* > 0.05 and recognition <15% in all strata). Robustness of tier assignments to alternative thresholds (60/20 and 80/10) is shown in Supplementary Note S1.

Benjamini–Hochberg false discovery rate (FDR) correction (α = 0.05) was applied separately to two pre-specified families of χ^2^ tests across age strata: 16 knowledge items and 6 information-source items; FDR-adjusted *p* values are reported alongside raw *p* values. Missing data were minimal. The awareness item and the knowledge battery were interviewer-administered, which limited item-level non-response: of the 233 aware respondents, 6 left at least one knowledge-battery item unanswered (treatment availability, *n* = 4; transmission routes and animal signs, *n* = 1 each), with no missing responses on the remaining items. Because unselected or unrecorded options were scored as incorrect in the binary composite, a complete composite knowledge score was available for all 233 aware respondents, and no participant was excluded from the descriptive or item-level analyses for missing knowledge data. In the adequate-knowledge logistic regression model, two aware respondents with missing covariate data were excluded by complete-case analysis (final *N* = 231).

### 2.5. Ethics

The study was conducted in accordance with the Declaration of Helsinki and received approval from the Balıkesir University Faculty of Medicine Health Research Ethics Committee (decision no. 2026/1-2; date 6 January 2026). Written informed consent was obtained from each participant prior to enrolment. No personally identifiable information was recorded, and electronic data were stored on password-protected institutional servers accessible only to the research team.

## 3. Results

### 3.1. Study Population and Overall Awareness

Of the 400 adults enrolled, 397 constituted the analytic sample after exclusion of three records lacking age and verification of four transcription-corrected age entries against original paper consent forms (Supplementary Figure S1). Mean age was 36.1 ± 15.8 years (median 29, range 18–82); 249 (62.7%) were male (Table 1). Reflecting the referral profile of a tertiary urban teaching hospital, 321 (80.9%) resided in urban districts, and 262 (66.0%) held a university or postgraduate degree. The most frequent occupational groups were student (142; 35.8%), worker (87; 21.9%), civil servant or professional (62; 15.6%), retiree (35; 8.8%), homemaker (33; 8.3%), and farmer or livestock handler (28; 7.1%). Active livestock involvement was reported by 60 participants (15.1%).

Overall, 233 of 397 respondents (58.7%) reported having heard of brucellosis prior to the survey. Awareness increased steadily with age: 51.8% (103/199) in 18–29 years, 57.6% (49/85) in 30–44 years, 69.7% (46/66) in 45–59 years, and 74.5% (35/47) in ≥60 years (Cochran–Armitage trend Z = 3.44, *p* < 0.001) (Figure 2A).

### 3.2. Composite Knowledge Score Among Aware Respondents

Among the 233 aware respondents, the mean composite knowledge score was 7.34 ± 4.25 out of 26 (median 7; IQR 4–10). The composite score declined across age strata (Figure 2A): 8.52 ± 4.50 in 18–29 years, 6.69 ± 3.75 in 30–44, 6.65 ± 3.83 in 45–59, and 5.94 ± 4.56 in ≥60 (Kruskal–Wallis *p* < 0.001; Spearman ρ with continuous age = −0.32, *p* < 0.001). The proportion meeting the adequate-knowledge threshold (composite ≥ 7 of 26) likewise declined: 68.0% in 18–29 to 34.3% in ≥60 (Cochran–Armitage Z = −3.98, *p* < 0.001) (Figure 2A). A bootstrap-derived exploratory Communication Efficiency Index, integrating awareness breadth and knowledge depth, yielded values clustered within a narrow band across all four strata (range 14.4–17.8%, with overlapping 95% confidence intervals; Supplementary Table S2), indicating that the four cohorts reached comparable total population-level literacy through compositionally different combinations of awareness and depth.

### 3.3. Item-Level Knowledge Profile by Age Stratum

Item-level analysis of the 16 substantive knowledge items revealed a non-uniform pattern across age strata (Table 2; Figure 2B). Two items showed uniformly high recognition across all age strata: raw dairy as a transmission route (82.9–89.8%; FDR *p* = 0.893) and treatment availability (79.6–82.9%; FDR *p* = 0.981).

Five items showed significant age-stratum differences after FDR correction, with substantially lower recognition in older respondents: fever as a clinical symptom (68.9% in 18–29 vs. 34.3% in ≥60; FDR *p* = 0.005); night sweats (48.5% vs. 14.3%; FDR *p* < 0.001); appetite loss (40.8% vs. 11.4%; FDR *p* = 0.013); contaminated meat as a transmission route (35.0% vs. 20.0%; FDR *p* = 0.007); and decreased milk yield as an animal sign (36.9% vs 8.6%; FDR *p* = 0.001).

The remaining items showed uniformly low recognition without significant age-stratum differences, including testis inflammation as an animal sign (2.9–5.8%) and inhalation as a transmission route (5.7–7.8%). Figure 2B illustrates the age-stratum recognition heatmap for representative items.

Complementary item-level Spearman rank correlations between continuous age and recognition, stratified by tier classification, are presented in Supplementary Table S3 and are concordant with the categorical FDR-adjusted findings reported above.

### 3.4. Information-Source Ecosystems by Age Stratum

Self-reported information sources among aware respondents differed by age (Table 3). Internet citation declined from 41.7% in 18–29 to 17.1% in ≥60 (FDR *p* = 0.003); healthcare-worker citation also declined (34.0% to 25.7%; FDR *p* = 0.003). Family or neighbor citation showed an opposite numerical trend (30.1% in 18–29 to 54.3% in ≥60) but did not survive FDR correction (FDR *p* = 0.115). Veterinarian, television or radio, and print media sources were not significantly differentiated across age strata after FDR correction.

### 3.5. Multivariable Adjusted Predictors of Awareness and Adequate Knowledge

In the multivariable logistic regression for awareness (*N* = 397), age ≥60 years was the strongest independent predictor after simultaneous adjustment for sex, education, residence, and livestock involvement (aOR 3.80, 95% CI 1.74–8.30; *p* < 0.001) (Table 4). Awareness also increased across intermediate strata (30–44: aOR 1.81, *p* = 0.055; 45–59: aOR 2.67, *p* = 0.008). University or postgraduate education (aOR 2.46, 95% CI 1.38–4.37; *p* = 0.002) and livestock involvement (aOR 2.35, 95% CI 1.13–4.89; *p* = 0.023) were independently associated with higher awareness; urban residence showed an inverse association (aOR 0.48, *p* = 0.030). Discrimination was modest (AUC = 0.668) and calibration excellent (Hosmer–Lemeshow *p* = 0.924).

**Figure 3:**
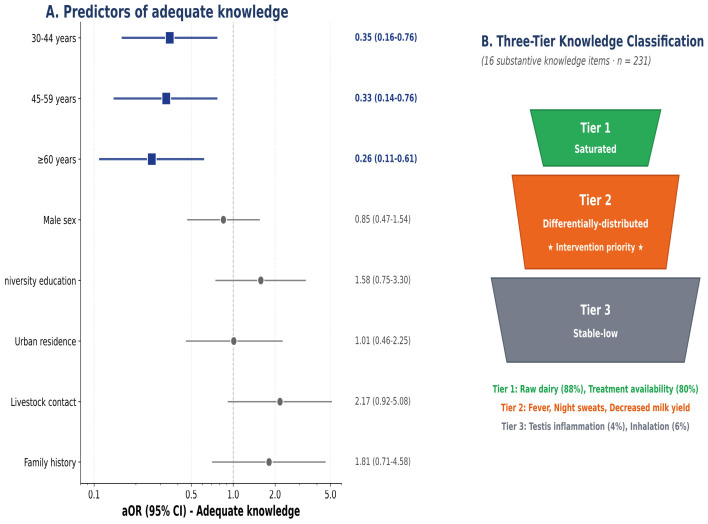
Multivariable predictors of adequate knowledge and the exploratory Three-Tier Knowledge Classification framework in 397 adults attending a tertiary teaching hospital in Balıkesir, Türkiye. (**A**) Forest plot of adjusted odds ratios with 95% confidence intervals from the multivariable logistic regression model for adequate knowledge among aware respondents (*N* = 231, complete-case for age stratification), illustrating the strong inverse association of age ≥60 years (aOR 0.26, 95% CI 0.11–0.61). (**B**) Three-Tier Knowledge Classification summarizing the FDR-corrected χ^2^ primary analysis of 16 substantive knowledge items: Tier 1 (saturated, FDR *p* > 0.05 with recognition >70%), Tier 2 (differentially distributed, FDR *p* ≤ 0.05; intervention priority), and Tier 3 (stable-low, FDR *p* > 0.05 with recognition <15%). The Three-Tier Classification and Communication Efficiency Index are exploratory data-summarizing constructs developed for this study and require external validation in independent samples.

In the model for adequate knowledge among aware respondents (*N* = 231; Table 5; Figure 3A), age ≥60 years independently predicted a 74% reduction in the odds of adequate knowledge (aOR 0.26, 95% CI 0.11–0.61; *p* = 0.002); the intermediate strata showed substantial inverse effects (30–44: aOR 0.35, *p* = 0.008; 45–59: aOR 0.33, *p* = 0.009). Discrimination (AUC = 0.696) and calibration (Hosmer–Lemeshow *p* = 0.595) were satisfactory.

A pre-specified sensitivity analysis excluding the 142 student participants preserved the awareness pattern (age ≥60 aOR 4.20, 95% CI 1.72–10.23; *p* = 0.002), supporting robustness beyond the medical-student-dominated youngest stratum (Supplementary Note S1).

### 3.6. Information Sources as a Partial Explanatory Channel

In a secondary covariate-adjusted analysis, the crude linear-regression coefficient of continuous age on composite knowledge score (−0.066 per year; 95% CI −0.098 to −0.033; *p* < 0.001) was reduced by 22.1% after joint adjustment for four information-source indicators (adjusted coefficient of −0.051 per year; *p* = 0.001). Citation of healthcare workers was independently associated with a higher composite score (+3.54 points, *p* < 0.001) and emerged as the strongest correlate among measured channels; veterinarian citation also showed a positive effect (+1.90 points; *p* = 0.024). Approximately one-fifth of the age-related difference in composite knowledge was thus statistically explained by differential information-source use, with the unexplained portion likely reflecting factors not captured by the measured indicators.

## 4. Discussion

### 4.1. Key Findings

In a single-center, hospital-based, cross-sectional convenience-sampled survey of 397 adults attending a tertiary teaching hospital in an endemic brucellosis zone of northwestern Türkiye, we documented that the age-stratified distribution of brucellosis knowledge among aware respondents is not uniform but item-level heterogeneous. Three findings constitute the principal descriptive contribution. First, awareness of brucellosis increases with age, while substantive item-level knowledge among the aware decreases with age, generating an inverse relationship between simple awareness and depth of correct knowledge. Second, the depth-of-knowledge gradient is heterogeneous at the item level: recognition of the unpasteurized-dairy transmission route and recognition of treatment availability are essentially uniform across all four age strata, while recognition of clinical symptoms (fever, night sweats, appetite loss) and veterinary signs (decreased milk yield) is substantially lower in older strata after FDR correction. Third, information-source ecosystems differ across age strata: younger respondents emphasized internet and healthcare-worker channels, while older respondents showed numerical (though not statistically significant after correction) reliance on interpersonal family and neighbor networks; covariate adjustment for these channels reduced the crude age coefficient on composite knowledge by 22.1%, leaving approximately three-quarters unexplained.

Throughout this manuscript, the terms “age-stratified heterogeneity” and “generational differences” describe cross-sectionally observed between-stratum differences, not within-individual temporal change; the separation of age, birth-cohort, and period effects is addressed in the Section 4.3. With this framing, the dominant dairy-transmission message showed uniformly high recognition across all adult age strata, consistent with decades of focused Turkish public health communication on unpasteurized dairy as the principal preventive target [3,8]. The Communication Efficiency Index further indicated that total population-level literacy was broadly similar across strata despite different breadth-depth compositions, with younger cohorts combining moderate awareness with richer substantive knowledge and older cohorts combining high awareness with thinner knowledge.

The age-related decline in clinical-symptom and veterinary-sign recognition was not attributable to education, residence, sex, or occupational livestock involvement: the pattern persisted after simultaneous adjustment for these covariates in the multivariable model. Sensitivity analysis excluding the medical-student-dominated youngest stratum preserved the inverse age gradient for awareness, indicating that the magnitude of the young-old gap among aware respondents is robust to student exclusion. In a secondary covariate-adjusted analysis (not a formal mediation analysis, given the cross-sectional design), joint adjustment for self-reported internet, family or neighbor, healthcare-worker, and veterinarian channels reduced the crude age coefficient on the composite score by 22.1%, with healthcare-worker citation emerging as the strongest independent positive correlate (+3.54 points on the 0–26 composite score; *p* < 0.001) and veterinarian citation showing a significant positive effect (+1.90 points; *p* = 0.024). This partial reduction is consistent with information-source composition being one contributor to the age gradient, but leaves approximately three-quarters of the crude age effect unexplained, indicating that other mechanisms likely also operate.

These findings align with the contemporary health literacy framework, in which functional health literacy (recognition of a threat) and interactive or critical health literacy (integration and application of information) are analytically distinguishable constructs [24,25]; psychometric validation of the European Health Literacy Survey Questionnaire in the Turkish population supports the applicability of this framework to our cultural setting [26]. In our cohort, older strata show high functional literacy (high awareness, high dairy-route recognition) but lower interactive literacy, while younger strata show the opposite pattern. Within Türkiye, earlier community-based seroprevalence studies have documented substantial *Brucella* exposure across age groups, including elderly populations in mid-Anatolia [20], rural communities in Central Anatolia [21], and rural Western Anatolia where the present study region is situated [22]. However, these earlier Turkish studies focused predominantly on serological exposure rather than on age-stratified item-level knowledge; the present analysis extends this body of work by characterizing how knowledge composition itself varies across the adult age spectrum within an endemic Turkish province. A comparable configuration of high awareness coupled with limited substantive knowledge has been reported across other brucellosis-endemic settings, including Iran [27], China [13], Saudi Arabia [14,28], Ethiopia [29], and Arab communities in Israel [30]; however, those studies characterized the gap largely at the aggregate or occupational-subgroup level, whereas our data localize it to specific item domains, namely clinical-symptom and veterinary-sign recognition, and resolve how it widens across the adult age spectrum, identifying where the deficit is concentrated rather than only that it exists. The generational divergence in information sources is consistent with the broader literature on age-related digital inequality [31]. A 2019 meta-analysis [12] of 79 brucellosis KAP studies confirmed that pooled awareness levels exceed substantive knowledge globally, but subgroup analyses by specific knowledge items and age strata have been rare. International KAP studies show variable awareness levels across endemic settings; a recent cross-sectional study from the Qassim region of Saudi Arabia reported a comparable moderate awareness level (56.8%) among 1244 adults, with significant variation by age group and residence area, paralleling the demographic heterogeneity we observed [32]. Large clinical case series describing brucellosis manifestations [33,34] emphasize that community recognition of clinical heterogeneity is essential for timely health-seeking behavior, and the Middle East has been identified as a region in which coordinated public awareness campaigns are a necessary complement to veterinary control efforts [35]. Our study contributes item-specific evidence from an endemic South Marmara setting paired with channel-specific source characterization.

Several alternative interpretations of these findings merit consideration. First, the uniformly high recognition of raw-dairy transmission across age strata may reflect not message saturation but item-level salience asymmetry: dairy transmission is a culturally embedded heuristic in Turkish endemic regions, frequently mentioned in everyday discourse and family-level food preparation narratives, whereas specific clinical symptoms require formal medical exposure to recall. Under this view, what we describe as “saturation” may in part reflect baseline cultural knowledge that exists independently of formal public-health communication. Discriminating between saturation-by-messaging and saturation-by-cultural-embeddedness would require measuring household-level exposure to public-health campaigns, which our study did not capture. Second, the inverse age gradient for adequate knowledge among aware respondents could partly reflect cohort-level differences in formal educational exposure rather than genuine attrition of brucellosis-specific knowledge over time; older Turkish cohorts experienced compulsory schooling at a time when zoonotic disease education was less formalized. The 22.1% reduction in the age-knowledge coefficient after adjustment for information-source use is consistent with channel mix being one mechanism, but the residual three-quarters likely reflects unmeasured cohort-educational and content-saturation effects. Third, the modest area-under-curve values for both multivariable models (AUC 0.668 for awareness, 0.696 for adequate knowledge) indicate that the measured demographic and exposure covariates explain only a modest proportion of between-individual variation, leaving substantial unexplained heterogeneity that future studies should address through richer measurement of media exposure, social network composition, and household-level health communication patterns. Fourth, social desirability bias inherent in face-to-face interviewing may have inflated awareness rates more in older cohorts (where brucellosis terminology is more culturally familiar) than in younger cohorts, contributing to the observed awareness gradient independently of true knowledge. These alternative interpretations are not mutually exclusive and likely operate jointly to produce the observed pattern.

These findings have direct relevance for the One Health control of brucellosis in endemic settings analogous to ours. As a paradigmatic zoonotic febrile illness with reservoir overlap among small and large ruminants, transmission through informal raw-dairy consumption, and concentrated occupational exposure among livestock-keepers, brucellosis sits squarely at the human–animal–environment interface that defines the operational scope of integrated One Health surveillance and control programmes endorsed by the WHO-WOAH-FAO Tripartite collaboration on neglected zoonotic diseases [36]. Embedding age-stratified, source-appropriate brucellosis communication into existing One Health platforms in Türkiye and in analogous low- and middle-income endemic settings, rather than addressing the human-health side in isolation, is more likely to be effective and sustainable, particularly in endemic provinces where livestock contact remains a defining demographic feature of the at-risk population.

Programmatic implications may be considered with appropriate caution given the limitations of the present sample. If the dominant raw-dairy message shows uniformly high recognition across age strata, further intensification of that same message may offer diminishing marginal returns; reallocation of communication effort toward less-recognized knowledge domains (clinical symptom recognition supporting timely health-seeking, animal-sign recognition supporting livestock-keeper vigilance) is a hypothesis worth testing in future intervention studies. A particularly notable finding is the consistently low veterinarian citation rate across all age strata (range 8.7–11.4%; FDR *p* = 0.960), suggesting that veterinary professionals contribute minimally to community-level brucellosis literacy in our sample. From a One-Health perspective, this may represent a missed structural opportunity: veterinarians possess specialized knowledge of zoonotic transmission and animal-side prevention but appear to be underutilized as a public-facing information channel. Strengthening veterinary outreach in livestock-keeping subpopulations is therefore a potentially high-yield, targeted intervention complementary to existing primary-care messaging; one concrete option would be structured zoonosis-briefing modules delivered by provincial veterinary services during routine herd-health visits and at livestock markets, co-developed with primary care so that animal-side and human-side messages remain consistent. Beyond this, the generational divergence in information-source ecosystems suggests that channel-specific delivery may be worth evaluating: digital and structured-encounter channels for younger cohorts where existing usage patterns favor these modes, and trusted interpersonal and primary-care channels for older cohorts where family-network-mediated information transfer appears more prominent. These public-health communication considerations represent the human-sector component of an integrated control strategy and should be aligned with sustained veterinary-sector interventions targeting the livestock reservoir, in keeping with the One Health framework for zoonotic disease control [37].

### 4.2. Implementation Hypotheses: Three Areas for Future Programmatic Evaluation

The three-tier knowledge classification (Figure 3B) and the breadth-depth invariance captured by the Communication Efficiency Index suggest three areas for future programmatic evaluation. These are presented as hypotheses generated by the present descriptive analysis, requiring confirmation in community-based and longitudinal studies before translation to formal policy.

**Hypothesis** **1:** *High-recognition message maintenance.*

For Tier 1 items (raw dairy transmission, treatment availability), recognition was uniformly high across all adult age strata. Marginal returns from further intensification of these specific messages are likely diminishing. A possible programmatic direction would be maintenance rather than amplification: continue existing messaging at current intensity through routine primary-care encounters, school health curricula, and standard provincial health-directorate materials, with redirection of any incremental communication budget away from these high-recognition targets toward less-recognized domains. In practice, maintenance could rely on low-cost embedded reminders, for example, standardized raw-dairy and treatment-availability messages incorporated into routine antenatal, childhood-vaccination, and chronic-disease visit materials, rather than dedicated stand-alone campaigns; periodic low-intensity reinforcement of this kind is generally sufficient to sustain already-saturated health messages.

**Hypothesis** **2:** *Differentially distributed message targeting.*

For Tier 2 items (clinical symptoms, including fever, night sweats, and appetite loss; the transmission route of contaminated meat; and the veterinary sign of decreased milk yield), substantial young–old recognition gradients persist after FDR correction. Possible age-stratified programmatic levers might include: targeted clinical-symptom recognition campaigns delivered through family-physician outreach in older adults aged 45 years and above (the strata showing the steepest deficits), and structured veterinary-sign awareness modules embedded in routine livestock-keeper interactions with provincial veterinary services. The covariate-adjustment analysis indicating that healthcare-worker channels carry the largest independent contribution to composite knowledge (+3.54 points; *p* < 0.001) is consistent with primary-care-based delivery being a high-yield channel, although this requires testing in randomized intervention studies.

**Hypothesis** **3:** *Channel-stratified delivery.*

The information-source ecosystem analysis demonstrates that younger cohorts emphasize digital and structured-encounter channels (internet 41.7% in 18–29 falling to 17.1% in ≥60), while older cohorts show numerical (though not statistically significant after FDR correction) reliance on family and neighbor networks. This generational divergence in channel ecosystems suggests that a single, uniform delivery strategy may be suboptimal. Possible channel-stratified pairings warranting evaluation include: for cohorts under 45 years, social-media-based public health campaigns with verified-source attribution; for cohorts 45 years and above, delivery through family-physician encounters, mobile veterinary services, and faith-community or neighbor-network mediated outreach where culturally appropriate. These hypotheses generate testable predictions for future cluster-randomized intervention studies of brucellosis communication strategies, but should not be interpreted as recommendations for immediate policy change without independent confirmation in community-based samples.

### 4.3. Limitations

Several important limitations warrant explicit consideration. First, and most consequentially, hospital-based convenience sampling at a tertiary teaching hospital substantially over-represents urban, university-educated, and student populations and under-represents the rural, older, less-educated populations in whom brucellosis incidence is highest in Türkiye [4]; 142 of 397 respondents (35.8%) reported student occupation, predominantly in the 18–29 stratum, and the smallest age stratum (≥60 years; *n* = 47) limits the precision of stratum-specific point estimates, particularly for low-prevalence Tier 3 items. The age-related awareness gradient documented here cannot be assumed to generalize to community-based or rural populations without independent replication; although our sensitivity analysis excluding all 142 students preserved and slightly strengthened the awareness gradient (≥60 aOR 4.20 vs. 3.80), the events-per-variable ratio in the non-student adequate-knowledge model fell to 8.6, and the magnitude of the young-old gap should therefore be interpreted as an upper bound. Second, all measurements were self-reported in a single face-to-face interview without clinical, behavioral, or serological cross-validation; social desirability bias is plausible given the interview format, and the awareness gradient could partly reflect cumulative lifetime exposure to brucellosis-related public-health messaging in older cohorts rather than current functional knowledge, a conceptual ambiguity that cannot be disentangled in a single-encounter cross-sectional design. Third, the study-specific questionnaire was not formally validated; construct and criterion validities and test-retest reliability were not assessed, and the acceptable internal consistency (Cronbach α = 0.822) is a post hoc estimate that does not substitute for prospective validation (Methods). The Three-Tier Knowledge Classification and Communication Efficiency Index are likewise exploratory analytic constructs developed for this study; their validity and reliability have not been established in independent samples.

Fourth, the cross-sectional design fundamentally precludes causal inference and does not allow separation of birth-cohort effects (older adults having received different educational exposures decades ago), period effects (secular changes in public-health messaging), and any genuine within-individual age-related change; the patterns described here represent differences between co-existing age strata at a single time point, not documented loss over time. The instrument was also developed for the Turkish context and has not been cross-culturally validated; comparisons with KAP studies from other regional and Central Asian endemic settings should account for instrument-specific differences. Fifth, the instrument did not distinguish between *Brucella melitensis* and *B. abortus* transmission sources; in Türkiye, where *B. melitensis* (small ruminants, raw goat and sheep dairy) is the predominant cause of human brucellosis, this conflation may obscure clinically and epidemiologically important differences in source recognition; future instruments should separate small-ruminant from bovine source recognition to enable species-specific intervention design. The observed pattern should therefore be interpreted as differential age-stratified knowledge distribution at a single time point in a hospital-based sample, not as documented loss over time or as representative of community-level knowledge structures.

Future research priorities follow directly from these limitations. First, a community-based replication using probability-based sampling (such as cluster random sampling at the village or neighborhood level) in fully endemic rural districts would test whether the age-stratified heterogeneity pattern documented here generalizes beyond the urban tertiary referral context. Second, a longitudinal panel design with the same individuals reassessed at 3–5-year intervals would help disentangle genuine age-related knowledge change from birth-cohort and period effects. Third, a cluster-randomized controlled trial of the Three-Tier framework, with interventions specifically targeting Tier 2 differentially distributed items in older strata, would directly evaluate the programmatic implications drawn here. Fourth, a linkage study connecting individual KAP responses to brucellosis seropositivity status (through Rose Bengal or tube agglutination testing in a subset of participants) would test whether the item-level knowledge gradients identified here translate to differential disease risk. Fifth, multi-center replication across other endemic Turkish regions, particularly Eastern and Southeastern Anatolia, where seroprevalence and livestock contact are higher, would establish geographic generalizability and identify region-specific variations in the age-stratified pattern. Sixth, structural equation modeling approaches, such as those applied in recent Chinese [13] and Saudi Arabian [14] studies, could complement the present descriptive analysis by formally modeling the K→A→P pathway in Turkish populations.

## 5. Conclusions

In this hospital-based sample from an endemic Turkish province, brucellosis knowledge was not uniformly distributed across the adult age spectrum. Recognition of the dominant unpasteurized-dairy transmission route and treatment availability was uniformly high across all age strata, reflecting decades of focused Turkish public-health communication. However, recognition of clinical symptoms (fever, night sweats, appetite loss) and veterinary signs (decreased milk yield) was substantially lower in older respondents, the very cohorts with the highest brucellosis exposure risk in Turkish endemic regions. The accompanying generational divergence in information-source ecosystems, with younger respondents emphasizing digital and healthcare-worker channels and older respondents relying more on interpersonal networks, demonstrates that uniform delivery strategies cannot equitably reach the full adult age spectrum.

These findings indicate that the next phase of brucellosis communication in endemic Turkish settings should integrate age-stratified, channel-specific reinforcement of less-recognized clinical and veterinary knowledge domains, particularly through the trusted healthcare-worker channels that emerged as the strongest correlate of adequate knowledge in our covariate-adjusted analysis. Embedding this approach into existing One Health platforms aligned with WHO-WOAH-FAO Tripartite guidance on neglected zoonotic diseases may meaningfully strengthen population-level prevention in endemic Turkish provinces and analogous low- and middle-income endemic settings, contributing to the broader global effort to reduce the burden of this neglected zoonotic disease.

These programmatic implications should be interpreted with appropriate caution. Given the hospital-based convenience sampling frame, which over-represents urban and university-educated populations, the non-validated study-specific instrument, and the single-center setting, community-based replication using probability-based sampling and a formally validated questionnaire is required before broader policy translation.

## Figures and Tables

**Table 1:** Sociodemographic characteristics and risk factors of study participants (*N* = 397).

Characteristic	Total (*N* = 397)	18–29 yr (*n* = 199)	≥60 yr (*n* = 47)
**Demographics**			
Age, mean ± SD (years)	36.1 ± 15.8	22.9 ± 2.6	66.4 ± 5.5
Male sex, *n* (%)	249 (62.7)	130 (65.3)	33 (70.2)
University education, *n* (%)d	262 (66.0)	169 (84.9)	11 (23.4)
Urban residence, *n* (%)	321 (80.9)	173 (86.9)	31 (66.0)
**Risk factors**			
Livestock involvement, *n* (%)	60 (15.1)	20 (10.1)	9 (19.1)
Family history of brucellosis, *n* (%) *	25 (10.7)	9 (8.7)	4 (11.4)
**Primary outcomes**			
Awareness of brucellosis, *n* (%)	233 (58.7)	103 (51.8)	35 (74.5)
Composite knowledge score, mean ± SD ^†^	7.34 ± 4.25	8.52 ± 4.50	5.94 ± 4.56

* Asked only to aware respondents (*n* = 233). ^†^ Among aware respondents (*n* = 233); range 0–26.

**Table 2:** Item-level recognition rates of 16 substantive knowledge items by age stratum among aware respondents (*n* = 233), with Benjamini–Hochberg FDR-corrected χ^2^ inference.

Knowledge Item	18–29 (*n* = 103)	30–44 (*n* = 49)	45–59 (*n* = 46)	≥60 (*n* = 35)	*p* (Raw)	*p* (FDR)
*Tier 1 (saturated)*						
Raw dairy as transmission route	88.3	89.8	89.1	82.9	0.769	0.893
Treatment availability	79.6	81.6	80.4	82.9	0.981	0.981
*Tier 2 (differentially distributed)*						
Fever (clinical symptom)	68.9	46.9	47.8	34.3	0.001	0.005
Night sweats	48.5	10.2	15.2	14.3	<0.001	<0.001
Appetite loss	40.8	22.4	28.3	11.4	0.002	0.013
Decreased milk yield (animal)	36.9	14.3	13.0	8.6	<0.001	0.001
Contaminated meat (transmission)	35.0	8.2	21.7	20.0	0.002	0.007
*Tier 3 (stable-low)*						
Inhalation as transmission route	7.8	10.2	4.3	5.7	0.747	0.893
Testis inflammation (animal)	5.8	2.0	0.0	2.9	0.275	0.481

Values are percentages. *p*-values from Pearson χ^2^ test across four age strata; FDR = Benjamini–Hochberg false discovery rate adjustment applied to 16 substantive knowledge items. Tier classifications were pre-specified before analysis.

**Table 3:** Information sources by age stratum among aware respondents (*n* = 233).

Information Source	18–29 (*n* = 103)	30–44 (*n* = 49)	45–59 (*n* = 46)	≥60 (*n* = 35)	*p* (FDR)
Internet/social media	41.7	20.4	15.2	17.1	0.003 *
Healthcare worker	34.0	6.1	17.4	25.7	0.003 *
Family/neighbor	30.1	42.9	43.5	54.3	0.115
Television/radio	20.4	10.2	13.0	22.9	0.485
Veterinarian	8.7	10.2	10.9	11.4	0.960
Print media	7.8	8.2	6.5	5.7	0.960

Values are percentages. *p*-values from Pearson χ^2^ test with Benjamini–Hochberg FDR correction across 6 information-source items. * FDR *p* ≤ 0.05.

**Table 4:** Multivariable logistic regression for predictors of brucellosis awareness (*N* = 397).

Predictor	aOR	95% CI	*p*
Age stratum (ref: 18–29 yr)			
30–44 yr	1.81	0.99–3.31	0.055
45–59 yr	2.67	1.29–5.54	0.008
≥60 yr	3.80	1.74–8.30	<0.001
Male sex (ref: female)	1.32	0.85–2.04	0.216
University education (ref: less)	2.46	1.38–4.37	0.002
Urban residence (ref: rural)	0.48	0.25–0.93	0.030
Livestock involvement (ref: none)	2.35	1.13–4.89	0.023

aOR = adjusted odds ratio; CI = confidence interval. Model AUC = 0.668; Hosmer–Lemeshow *p* = 0.924; maximum VIF = 4.34.

**Table 5:** Multivariable logistic regression for predictors of adequate knowledge (composite score ≥ 7) among aware respondents (*N* = 231, complete-case for age stratification).

Predictor	aOR	95% CI	*p*
Age stratum (ref: 18–29 yr)			
30–44 yr	0.35	0.16–0.76	0.008
45–59 yr	0.33	0.14–0.76	0.009
≥60 yr	0.26	0.11–0.61	0.002
Male sex (ref: female)	0.85	0.47–1.54	0.596
University education (ref: less)	1.58	0.75–3.30	0.227
Urban residence (ref: rural)	1.01	0.46–2.25	0.974
Livestock involvement (ref: none)	2.17	0.92–5.08	0.075
Family history of brucellosis	1.81	0.71–4.58	0.214

aOR = adjusted odds ratio; CI = confidence interval. Adequate knowledge = composite score ≥ 7 (sample median). Model AUC = 0.696; Hosmer–Lemeshow *p* = 0.595.

## Data Availability

The original contributions presented in the study are included in the article and Supplementary Material. The de-identified individual-participant dataset (CSV) and the complete Python analysis code (Jupyter notebook) used to generate all results, tables, and figures are available from the corresponding author upon reasonable request, subject to institutional data-sharing policy and ethics committee approval.

## Supplementary Materials

The following supporting information can be downloaded at: https://www.mdpi.com/article/10.3390/tropicalmed11070185/s1, Figure S1: Participant flow diagram; Note S1: Detailed statistical methodology and sensitivity analyses; Table S1: Item-total correlations for the 26-item composite knowledge score; Table S2: Communication Efficiency Index (CEI) bootstrap confidence intervals (1000 resampling iterations); Table S3: Item-level Spearman correlations between continuous age and recognition, by tier; STROBE checklist for cross-sectional studies.

## Abbreviations

The following abbreviations are used in this manuscript:

VIF	Variance inflation factor
STROBE	Strengthening the Reporting of Observational Studies in Epidemiology
OR	Odds ratio
LR	Logistic regression
KAP	Knowledge, attitudes, and practices
IRB	Institutional Review Board
FDR	False discovery rate
CRediT	Contributor Roles Taxonomy
CI	Confidence interval
CEI	Communication Efficiency Index
CC	Complete case
BCa	Bias-corrected and accelerated
aOR	Adjusted odds ratio
