# Supplementary material for: Age-Stratified Heterogeneity of Brucellosis Awareness and Knowledge Among Hospital-Attending Adults in an Endemic Turkish Province: A Single-Center Cross-Sectional KAP Study
Source: Trop Med Infect Dis. 2026 Jul 6;11(7):185. doi: 10.3390/tropicalmed11070185 (PMC13417325; doi:10.3390/tropicalmed11070185)
Supplement: Supplementary file 1 [file tropicalmed-11-00185-s001.zip › STROBE_Checklist.pdf]

STROBE Statement Checklist

Cross-sectional studies: instrument development and reliability

This manuscript reports a cross-sectional KAP study and adheres to the STROBE Statement (Strengthening the Reporting of Observational Studies in Epidemiology). The 22-item checklist is provided below with corresponding page and section references in the manuscript.

Item No.	Recommendation	Reported on	Status
**1a**	Indicate the study design with a commonly used term in the title or abstract	Title; Abstract (page 1)	✓
**1b**	Provide an informative and balanced summary of objectives, methods, results, conclusion	Abstract (page 1)	✓
**2**	Background/rationale: Explain scientific background and rationale	Introduction (pages 2-3)	✓
**3**	Objectives: State specific objectives, including pre-specified hypotheses	Introduction, Objectives (page 3)	✓
**4**	Study design: Present key elements of study design early in the paper	Methods 2.1 (page 4)	✓
**5**	Setting: Describe setting, locations, dates, recruitment, exposure, follow-up, data collection	Methods 2.1 (page 4); Figure 1	✓
**6**	Participants: Eligibility criteria, sources, methods of selection	Methods 2.1 (pages 4-5); Figure S1	✓
**7**	Variables: Define all outcomes, exposures, predictors, confounders	Methods 2.2 (page 5)	✓
**8**	Data sources/measurement: Methods of assessment for each variable	Methods 2.2-2.3 (pages 5-6)	✓
**9**	Bias: Describe efforts to address potential sources of bias	Methods 2.4 (pages 6-7); Discussion 4.3 (page 16)	✓
**10**	Study size: Explain how study size was arrived at	Methods 2.1 (page 5)	✓
**11**	Quantitative variables: Explain handling and grouping in analysis	Methods 2.2 (page 5); Methods 2.4 (pages 6-7)	✓
**12a**	Statistical methods: Describe all statistical methods including those for confounding	Methods 2.4 (pages 6-7)	✓
**12b**	Statistical methods: Describe methods used to examine subgroups and interactions	Methods 2.4 (page 7); Table 4	✓
**12c**	Statistical methods: Explain how missing data were addressed	Methods 2.4 (page 7)	✓
**12d**	Statistical methods: Cross-sectional study: describe analytical methods accounting for sampling strategy	Methods 2.4 (page 7)	✓
**12e**	Statistical methods: Describe sensitivity analyses	Methods 2.4 (page 7); Supplementary Note S1	✓
**13**	Participants (Results): Numbers at each stage, reasons for non-participation	Results 3.1 (page 8); Figure S1	✓

**14**	Descriptive data: Characteristics of study participants	Results 3.1 (page 8); Table 1	✓
**15**	Outcome data: Numbers of outcome events or summary measures	Results 3.2-3.3 (pages 8-10); Tables 2-3	✓
**16**	Main results: Estimates and confidence intervals; consider periods, exposure categories	Results 3.2-3.6 (pages 8-11); Tables 4-5; Figures 2-3	✓
**17**	Other analyses: Subgroups, interactions, sensitivity analyses	Results 3.4-3.6 (pages 10-11); Table S3; Supplementary Note S1	✓
**18**	Key results: Summarize key results with reference to study objectives	Discussion 4.1 (page 11)	✓
**19**	Limitations: Discuss limitations including bias, imprecision, generalizability	Discussion 4.3 (pages 15-16)	✓
**20**	Interpretation: Cautious overall interpretation considering context, biases	Discussion 4.1-4.2 (pages 11-15)	✓
**21**	Generalizability: External validity	Discussion 4.3 (page 16)	✓
**22**	Funding: Source of funding and role of funders	Funding statement (page 17)	✓

Note: Page numbers refer to the manuscript file. The STROBE Statement was developed and published by von Elm E, Altman DG, Egger M, Pocock SJ, Gøtzsche PC, Vandenbroucke JP. The Strengthening the Reporting of Observational Studies in Epidemiology (STROBE) statement: guidelines for reporting observational studies. Lancet. 2007;370(9596):1453-1457. Original checklist: http://www.strobe-statement.org

Compliance Summary: All 22 STROBE items (with sub-items) are addressed in the manuscript. Total: 27/27 items reported.
