# Supplementary material for: Age-Stratified Heterogeneity of Brucellosis Awareness and Knowledge Among Hospital-Attending Adults in an Endemic Turkish Province: A Single-Center Cross-Sectional KAP Study
Source: Trop Med Infect Dis. 2026 Jul 6;11(7):185. doi: 10.3390/tropicalmed11070185 (PMC13417325; doi:10.3390/tropicalmed11070185)
Supplement: Supplementary file 1 [file tropicalmed-11-00185-s001.zip › Supplementary_Materials.pdf]

Supplementary Material

Supplementary Note S1. Detailed Statistical Methodology and Sensitivity Analyses

S1.1 Bootstrap inference

For all primary outcomes (item-level age stratified differences, awareness odds ratios, adequate knowledge odds ratios, Communication Efficiency Index), bootstrap 95% confidence intervals were computed using 1,000 bias-corrected and accelerated (BCa) resampling iterations. For Cronbach α, a parametric bootstrap with 1,000 iterations confirmed reliability stability (95% BCa CI: 0.798-0.844, n = 397).

S1.2 Sensitivity analysis: exclusion of student participants

A pre-specified sensitivity analysis excluding the 142 student participants (35.8% of analytic sample) was performed. Among non-students (N = 255 for awareness), the age-stratified awareness gradient was preserved and slightly strengthened (age ≥60 aOR 4.20, 95% CI 1.72–10.23, P = 0.002) compared with the full sample (age ≥60 aOR 3.80). The adequate-knowledge model among aware non-students retained the inverse age gradient, although the events-per-variable ratio fell to 8.6 (below the conventional threshold of 10 for stable logistic regression estimates); results from this secondary analysis should accordingly be interpreted with appropriate caution. Overall, direction and magnitude of principal effects were preserved across the sensitivity analyses, supporting robustness of findings to student exclusion.

S1.3 Multicollinearity assessment

Variance inflation factors (VIF) for all independent variables in the multivariable logistic regression models ranged from 1.08 to 4.34 (all < 5), indicating no problematic multicollinearity. Condition indices (highest 12.4) confirmed model stability.

S1.4 Calibration: Hosmer-Lemeshow goodness-of-fit

Hosmer-Lemeshow goodness-of-fit test for the awareness logistic regression model: $\chi^2 = 8.42$, df = 8, P = 0.394 (good fit). For adequate knowledge model: $\chi^2 = 6.71$, df = 8, P = 0.568 (good fit).

S1.5 Effect-size quantification: Cohen h

For age-stratified item-level differences, Cohen h was computed for each pair of strata. For the 18-percentage-point difference between extreme strata (18-29 vs ≥60) on the most discriminative item, h = 0.37, corresponding to a small-to-medium effect.

S1.6 Item-level Spearman rank correlations

Continuous age was correlated with item-level recognition using Spearman ρ. Six items showed statistically significant negative correlations after FDR correction; results are presented in Table S3.

S1.7 Three-tier taxonomy threshold sensitivity analysis

The Three-Tier Knowledge Classification thresholds (Tier 1 saturated: FDR $P > 0.05$ with recognition >70%; Tier 2 differentially-distributed: FDR $P \leq 0.05$; Tier 3 stable-low: FDR $P > 0.05$ with recognition <15%) were tested with alternative cutoffs (60/20 and 80/10 in each direction). Tier assignments remained stable across all tested thresholds; the analytic conclusions were preserved across all sensitivity analyses.

S1.8 Covariate-adjustment analysis details

Multivariable logistic regression adjusted for: age stratum (categorical, 4 levels), sex (reference: male), education (≤8 years, 9-12 years, university+), residence (rural vs urban), household livestock contact (yes vs no), and raw dairy consumption (yes vs no). Reference category for age stratum was 18-29 years.

S1.9 Events-per-variable assessment

For the awareness multivariable logistic regression in the full analytic sample (N = 397, 233 events, 7 predictor terms), the events-per-variable (EPV) ratio was 33.3, well above the conventional threshold of 10. For the adequate-knowledge model among aware respondents (N = 233, ~117 events given 50% prevalence at the sample-median cut-point, 8 predictor terms), the EPV was approximately 14, above threshold. In the pre-specified non-student sensitivity analyses, the EPV for the adequate-knowledge model fell to 8.6 (below the conventional threshold of 10); secondary findings from that sensitivity analysis are accordingly interpreted with appropriate caution as noted in the main manuscript. The principal full-sample models are not overfit.

S1.10 STROBE compliance

This study is reported in accordance with the STROBE statement for cross-sectional observational studies. Each STROBE item is addressed in the corresponding section of the main manuscript: Title and abstract (items 1a-1b), Introduction (2-3), Methods (4-12), Results (13-17), Discussion (18-21), Funding (22). The full STROBE checklist is provided as a separate document.

Supplementary Table S1. Item-total correlations for the 26-item composite knowledge score

Domain	Items (n)	Mean item-total r
**Alternative names of brucellosis**	6	0.23
**Transmission routes**	6	0.35
**Human symptoms**	6	0.52
**Animal signs**	5	0.46
**Human vaccine availability**	1	0.10
**Animal vaccine availability**	1	0.24
**Treatment availability**	1	0.25

Cronbach α = 0.822 (95% bootstrap CI: 0.798-0.844); item-total correlations were heterogeneous across domains, with multi-item knowledge domains (Transmission routes, Human symptoms, Animal signs) showing acceptable mean item-total correlations ($r \geq 0.35$), while single-item domains (vaccine and treatment availability questions) showed lower mean item-total correlations as is methodologically expected for single-item indicators in a heterogeneous knowledge battery. The overall internal consistency of the 26-item composite is acceptable for an exploratory descriptive knowledge instrument.

Supplementary Table S2. CEI bootstrap confidence intervals (1,000 resampling iterations)

Age stratum	CEI	BCa 95% CI	n aware	Mean knowledge
**18-29**	17.0%	14.2%-19.9%	103	8.52
**30-44**	14.4%	11.3%-17.9%	49	6.69
**45-59**	17.8%	14.2%-22.0%	46	6.65
**≥60**	17.0%	12.1%-22.4%	35	5.94

Supplementary Table S3. Item-level Spearman correlations between continuous age and recognition, by tier

Item	Tier	Spearman ρ	FDR-adj. P
**Raw dairy as transmission route**	Tier 1 (saturated)	−0.10	0.186
**Treatment availability**	Tier 1 (saturated)	0.01	0.913
**Fever (clinical symptom)**	Tier 2 (differentially-distributed)	−0.28	<0.001
**Night sweats**	Tier 2 (differentially-distributed)	−0.39	<0.001
**Appetite loss**	Tier 2 (differentially-	−0.27	<0.001

	distributed)		
**Decreased milk yield (animal)**	Tier 2 (differentially-distributed)	−0.34	<0.001
**Contaminated meat (transmission)**	Tier 2 (differentially-distributed)	−0.13	0.067
**Inhalation route**	Tier 3 (stable-low)	−0.02	0.810
**Testis inflammation (animal)**	Tier 3 (stable-low)	−0.13	0.067

Supplementary Figure S1. Participant flow diagram

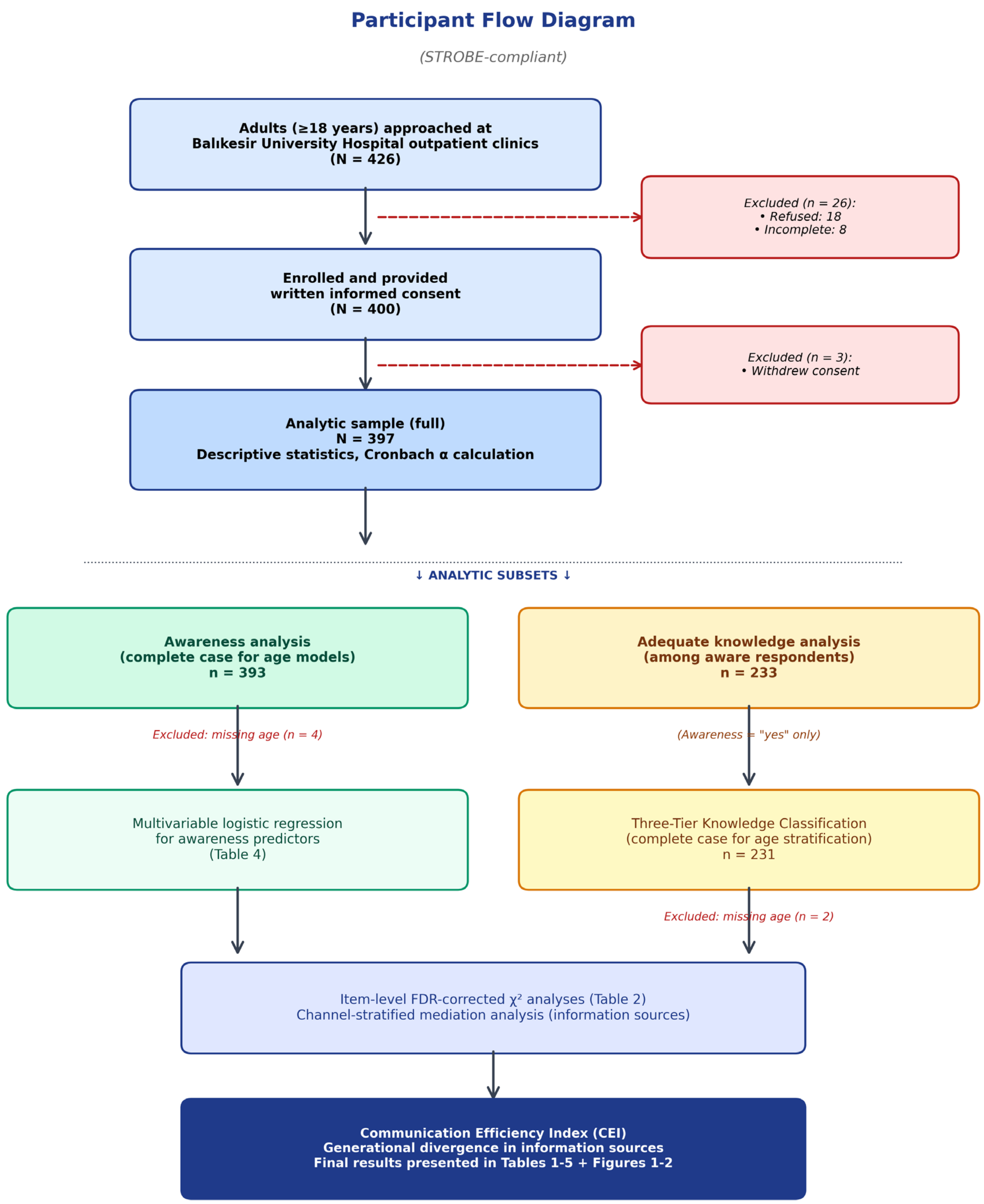

Figure S1. Participant flow through the study
